# A data-driven approach to estimating the number of clusters in hierarchical clustering

**DOI:** 10.12688/f1000research.10103.1

**Published:** 2016-12-01

**Authors:** Antoine E. Zambelli

**Affiliations:** 1 Quantech Solutions LLC, San Rafael, CA, USA

**Keywords:** Clustering, Hierarchy, Dendrogram, Gene Expression, Empirical

## Abstract

DNA microarray and gene expression problems often require a researcher to perform clustering on their data in a bid to better understand its structure. In cases where the number of clusters is not known, one can resort to hierarchical clustering methods. However, there currently exist very few automated algorithms for determining the true number of clusters in the data. We propose two new methods (mode and maximum difference) for estimating the number of clusters in a hierarchical clustering framework to create a fully automated process with no human intervention. These methods are compared to the established elbow and gap statistic algorithms using simulated datasets and the Biobase Gene ExpressionSet. We also explore a data mixing procedure inspired by cross validation techniques. We find that the overall performance of the maximum difference method is comparable or greater to that of the gap statistic in multi-cluster scenarios, and achieves that performance at a fraction of the computational cost. This method also responds well to our mixing procedure, which opens the door to future research. We conclude that both the mode and maximum difference methods warrant further study related to their mixing and cross-validation potential. We particularly recommend the use of the maximum difference method in multi-cluster scenarios given its accuracy and execution times, and present it as an alternative to existing algorithms.

## 1 Introduction

Hierarchical clustering analysis (HCA) is an extensively studied field of unsupervised learning. Very useful in dimensionality reduction problems, we will study ways of using this clustering method with the aim of reducing (or removing) the need for human intervention.

The problem of human intervention stems from the fact that HCA is used when the correct number of clusters in a dataset is not known (otherwise we might use, for example, K-means). While the ability to cluster data with an unknown number of clusters is a powerful one, a researcher often needs to interpret the results - or cutoff the algorithm - to recover a meaningful cluster number. While our work was prompted by DNA microarray analysis and gene expression problems, these methods can be applied to general hierarchical clustering scenarios. Specifically, we analyze different existing automated methods for cutting off HCA and propose two new ones.

In Section 2 we discuss background material on HCA and the existing methods and in Section 3 we present some technical details on these methods and introduce our own. Section 4 contains results on simulated and actual data, and Section 5 examines data sampling procedures to improve accuracy.

## 2 Background

Hierarchical clustering, briefly, seeks to pair up data points that are most similar to one another. With the agglomerative (or bottom-up) approach, we begin with *N* data points forming singleton clusters. For each point, we measure the distance between it and its *N* − 1 neighbors. The pair with the shortest distance between the two points is taken to form a new cluster. We then look at the distance between the *N* − 2 points remaining and the newly formed cluster, and again pair off the two points with the shortest distance (either adding a data point to our 2-cluster, or forming another cluster from two new data points). This process is repeated until there is a single cluster with *N* points (regardless of the absolute distance between points).

Naturally, this is a very good dimensionality reduction algorithm. Unfortunately, it continues until the data is flattened to 1 dimension. In cases where there are *n* ≥ 2 clusters, this is problematic.

**Figure 1.**
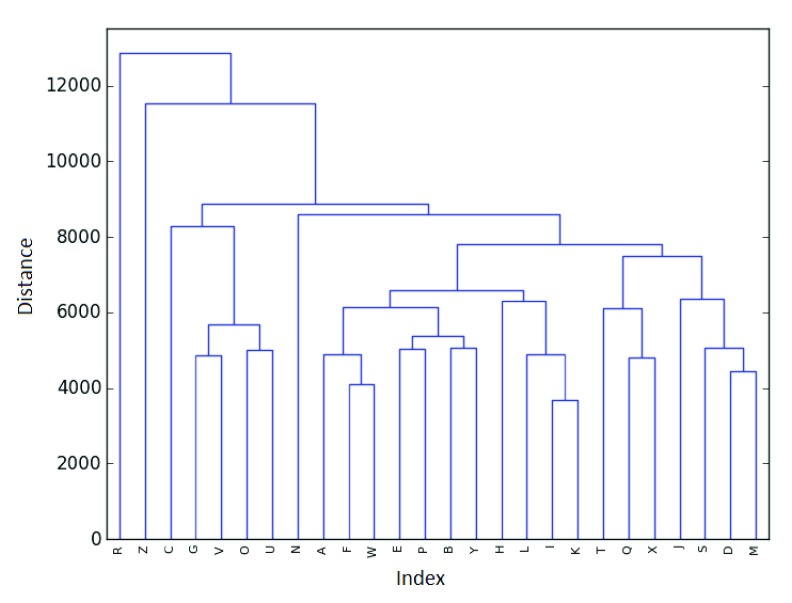
Example dendrogram from 26 data points.

The results of a HCA are often expressed as a dendrogram, a tree-like graph that contains vital information about the distances measured in the clustering and the pairings generated. An example of a dendrogram is shown in Figure 1. Briefly, horizontal lines denote pairings, and the height of those lines represent the distance that needs to be bridged in order to cluster the points together. That is, the smaller the height (or jump) of a pairing, the closer the points were to begin with.

Our goal is to find a way to stop the algorithm from arbitrarily flattening our data to 1 dimension. If another run would cluster two very dissimilar points together, it likely isn’t a scientifically sound choice. In that case we would stop the algorithm and keep the cluster structure built up to that point.

### 2.1 Existing alternatives

The problem of cutting off a dendrogram is one that researchers encounter often, but there are no reliable automated methods for doing so 1. Often, the gap statistic is the only proposed automated method, as in 1. As such, many researchers inspect the finished dendrogram and manually select a cutoff point, based on their own judgment. Apart from the obviously slow nature of this exercise, there is also the question of human error to consider - as well as bias. In cases where the cutoff is not easily determined, two different researchers may arrive at different conclusions as to the correct number of clusters - which could both be incorrect. Algorithmic approaches aim to eliminate this, and range from simpler methods to more complex ones. An excellent summary of existing methods is given in 2, which is referenced in 3.

The latter, more importantly, develops the gap statistic. We present the technical aspects in Section 3, but quickly discuss some properties here. First, the gap statistic is one of few methods that is capable of accurately estimating single clusters (in the case where all our data belongs to one cluster), a situation often undefined for other methods ^1^. While it is rather precise overall, it requires the use of a “reference distribution”, which must be chosen by the researcher. In 3, the authors put forward that the uniform distribution is in fact the best choice for unimodal distributions. A powerful result, it is still limited in other cases, and thus many researchers still take the manual approach. However, it generally outperforms other complex methods (see 3) and, as such, we focus on the gap statistic.

On the other side of the complexity spectrum, are variants of the “elbow method". The elbow method explains the variance in data as a function of the number of clusters assigned. The more clusters assigned, the more variance that can be explained. However, as we add more clusters we begin to get diminishing returns and each new cluster explains less and less of the variance - we choose the point where returns begin to diminish as the number of clusters. A variant of this method, often applied to dendrograms, looks for the largest acceleration of distance growth ^4^. While this method is very flexible, it cannot handle the single-cluster case ^4^.

## 3 Approaches

We look at both the elbow method variant and the gap statistic, as well as our own 2 methods. While there are many other methods to compare to, the gap statistic is quite representative of a successful (if more complex) solution - and tends to outperform other known methods ^3^. The elbow method is representative of the more accepted simple approaches. In all tests considered in this paper, an agglomerative hierarchy, with average linkage and euclidean distance measure is used.

### 3.1 Gap statistic

The gap statistic is constructed from the within-cluster distances, and comparing their sum to the expected value under a null distribution. Specifically, as given in 3, for *r* clusters *C _r_*

$$Gap_{n}(k)=E_{n}^{*}[\text{log}\;W_{k}]-\text{log}\;W_{k}\;(1)$$

where, with *n _r_* = *|C _r_|*,

$$W_{k}=\sum_{r=1}^{k}\frac{1}{2n_{r}}D_{r}=\sum_{r=1}^{k}\frac{1}{2n_{r}}\sum_{i,i^{\prime }\in C_{r}}d_{ii^{\prime }}\;(2)$$

That is, we are looking at the sum of the within-cluster distances *d*, across all *r* clusters *C _r_*. Computationally, we estimate the gap statistic and find the number of clusters to be (as per 3)

$${\hat{k}}_{G}=\text{smallest}\;k|Gap(k)\geq Gap(k+1)-s_{k+1}\;(3)$$

where *s _k_* is the standard error from the estimation of *Gap*( *k*). As mentioned ^3^, considers both a uniform distribution approach and a principal component construction. In many cases, the uniform distribution performs better, and this is used here.

### 3.2 Elbow method

This variant of the elbow method, which looks at the acceleration, is seen in 4. A straightforward method, we simply look at the acceleration in jump sizes. So given the set of distances from our clustering { *d*_1_, … , *d _N_*}, the acceleration can be written as

$$\left\{d_{3}-2d_{2}-d_{1},\cdots ,d_{N}-2d_{N-1}-d_{N-2}\}.\;(4\right)$$

We choose our number of clusters as the jump with the highest acceleration, giving us

$${\hat{k}}_{E}=N+2-\underset{i\in [3,N]}{\text{arg}\;\text{max}}\;\{d_{i}-2d_{i-1}-d_{i-2}\}.\;(5)$$

While very simple and very fast, this method will never find the endpoints, ie, the *N* singleton clusters and the single *N*-element cluster cases.

### 3.3 Mode

The first method we propose is based on the empirical distribution of jump sizes. Specifically, we use the mode of the distribution *D* = { *d*_1_, … , *d _N_*}, denoted *D̂*, adjusted by the standard deviation ( *σ _D_*). Our motivation is that the most common jump size likely does not represent a good cutoff point, and we should consider a higher jump threshold. As such, we take the number of clusters to be

$${\hat{k}}_{M}=\hat{D}+\alpha \sigma_{D},\;(6)$$

where *α* is a parameter that can be tuned. Naturally, tuning *α* would require human intervention or a training dataset. As such, we set it to 3 arbitrarily for a first look at the method’s viability.

### 3.4 Maximum difference

Our second method is even simpler, but is surprisingly absent from the literature. Inspired by the elbow method, we look at the maximum jump difference - as opposed to acceleration. Our number of clusters is then given by

$${\hat{k}}_{D}=N+2-\underset{i\in [2,N]}{\text{arg}\;\text{max}}\;\{d_{i}-d_{i-1}\}.\;(7)$$

This method shares the elbow method’s drawback that it cannot solve the single cluster case (though it can handle singleton clusters), but we thought it prudent to examine as the literature seemed to focus on acceleration and not velocity.

## 4 Results

We present results of simulations on several numbers of true clusters, drawn from a 2-dimensional normal distribution. Each cluster is comprised of 100 samples. We are most interested in tracking the success rate and the *error size given an incorrect estimate*. That is, how often can we correctly estimate the number of clusters *k* and when we can’t, by how much are we off? Formally, this is given by

$$S_{X}=\frac{\left|T\right|}{n},\;\text{with}\;T={\left\{j|{\hat{k}}_{X}^{\left(j\right)}=k\right\}}_{j=1}^{n}\;(8)$$

and

$$E_{X}=\frac{1}{\left|S\right|}\sum_{x\in S}x,\;\text{with}\;S={\left\{\left|k-{\hat{k}}_{X}^{\left(j\right)}\right||k\neq {\hat{k}}_{X}^{\left(j\right)}\right\}}_{j=1}^{n}.\;(9)$$

### 4.1 Simulated data

**Figure 2.**
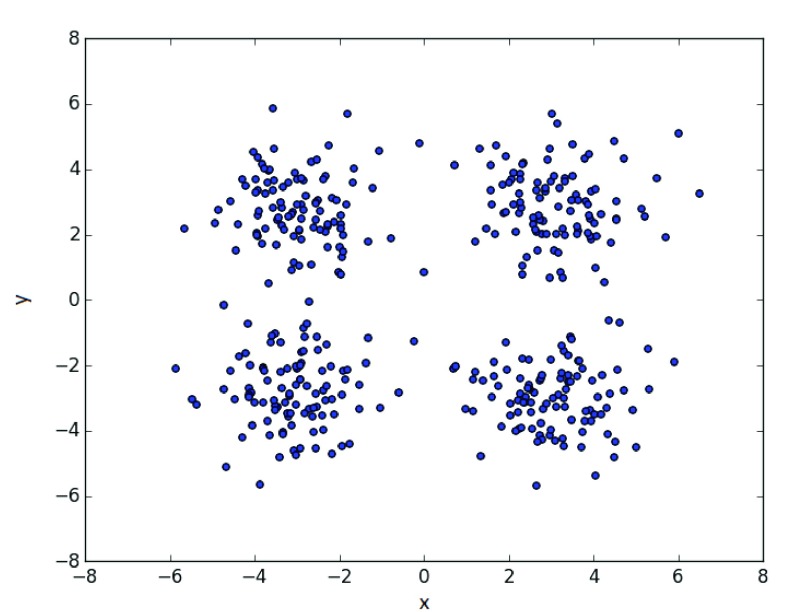
Sample clusters of 100 points each.

The data used was drawn from a standard normal distribution, with cluster centers at (−3, −3), (3, 3), (−3, 3), (3, −3), shown in Figure 2. In the case of 1 cluster, the first is taken, for 2 clusters, the first two, and so on. We present the results of the methods on *n* = 200 simulations below in Table 1– Table 3, with the best results in bold.

**Table 1.** Average cluster numbers over *n* = 200 runs.

*k*	k^¯E	k^¯G	k^¯D	k^¯M
1	3.720	**1.110**	2.965	4.505
2	**2.000**	**2.000**	**2.000**	2.645
3	3.050	3.050	**3.015**	4.725
4	3.915	4.060	**4.010**	6.280

**Table 2.** Success rate over *n* = 200 runs.

*k*	*S _E_*	*S _G_*	*S _D_*	*S _M_*
1	0.000	**0.910**	0.000	0.000
2	**1.000**	**1.000**	**1.000**	0.465
3	0.955	0.960	**0.985**	0.100
4	0.920	0.940	**0.990**	0.055

**Table 3.** Average error size (when wrong) over *n* = 200 runs.

*k*	*E _E_*	*E _G_*	*E _D_*	*E _M_*
1	2.720	**1.222**	1.965	3.505
2	**0.000**	**0.000**	**0.000**	1.206
3	1.111	1.250	**1.000**	1.917
4	1.688	**1.000**	**1.000**	2.413

As shown, the best performing methods so far are the gap statistic and, surprisingly, the maximum difference.

The maximum difference method has a near perfect success rate on this simple example, besting the gap statistic in most areas. As noted though, it suffers from the same problem as the elbow method in that it cannot handle the single-cluster case. It is our recommendation that if the reader suspects their data may be a single cluster, they should consider the gap statistic method. Note, however, that it is much more computationally intensive. As measured by Python’s timeit function, by a factor of ~ 50000.

### 4.2 Different cluster distributions

To get a better sense for the behavior of these methods, we look at clusters drawn from normal distributions with different parameters. For the clusters centered at (−3, −3), (3, 3), (−3, 3), (3, −3), we scale the standard deviations to use, respectively: _2_, _2_, 2 _2_, 0.5 _2_. Hopefully, these different distributions will stress the methods, highlight any weaknesses, and likely serve as a better proxy for real data. The results are detailed in Table 4– Table 6.

**Table 4.** Average cluster numbers over *n* = 200 runs.

*k*	k^¯E	k^¯G	k^¯D	k^¯M
1	3.710	**1.100**	3.035	4.450
2	**2.000**	**2.000**	**2.000**	2.625
3	3.170	**3.000**	3.060	6.055
4	3.940	4.140	**4.005**	5.780

**Table 5.** Success rate over *n* = 200 runs.

*k*	*S _E_*	*S _G_*	*S _D_*	*S _M_*
1	0.000	**0.910**	0.000	0.000
2	**1.000**	**1.000**	**1.000**	0.475
3	0.840	**1.000**	0.920	0.005
4	0.955	0.880	**0.995**	0.085

**Table 6.** Average error size (when wrong) over *n* = 200 runs.

*k*	*E _E_*	*E _G_*	*E _D_*	*E _M_*
1	2.710	**1.111**	2.035	3.450
2	**0.000**	**0.000**	**0.000**	1.190
3	1.063	**0.000**	1.000	3.070
4	1.778	1.167	**1.000**	1.945

In this more complicated case, we see similar results. The 3-cluster case (in this example) seems problematic for our method and the elbow method. The gap statistic once again performs well for the single-cluster scenario, but shows some weakness at 4 clusters. Overall, it seems that for *k* = 2, 3, 4 clusters, the maximum difference method at the very least is equal to the gap statistic, and improves on it in certain cases.

### 4.3 Different cluster separations

**Figure 3.**
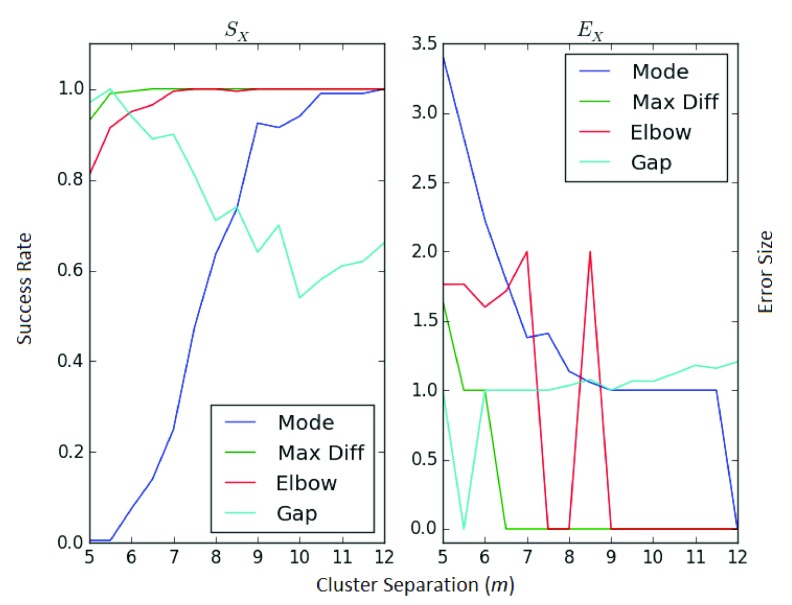
Tracking for varying distances.

Returning to the equal-distribution 4-cluster problem, we now look at how the metrics evolve the distance between the clusters is increased. On the *x*-axis in Figure 3, a value of *m* corresponds to a cluster arrangement with coordinates: (− *m/*2, − *m/*2), ( *m/*2, *m/*2), (− *m/*2, *m/*2), ( *m/*2, − *m/*2). We expect all methods to perform better as the clusters drift further apart, since they are then more distinct.

This is indeed the case for the elbow, maximum difference and mode methods, which converge to a success rate of 1 and an error size of 0 (note some increased stability in the maximum difference). However, the gap statistic appears to do worse as the clusters separate - which could point to some underlying issues and should be explored more fully.

### 4.4 Biobase set

**Figure 4.**
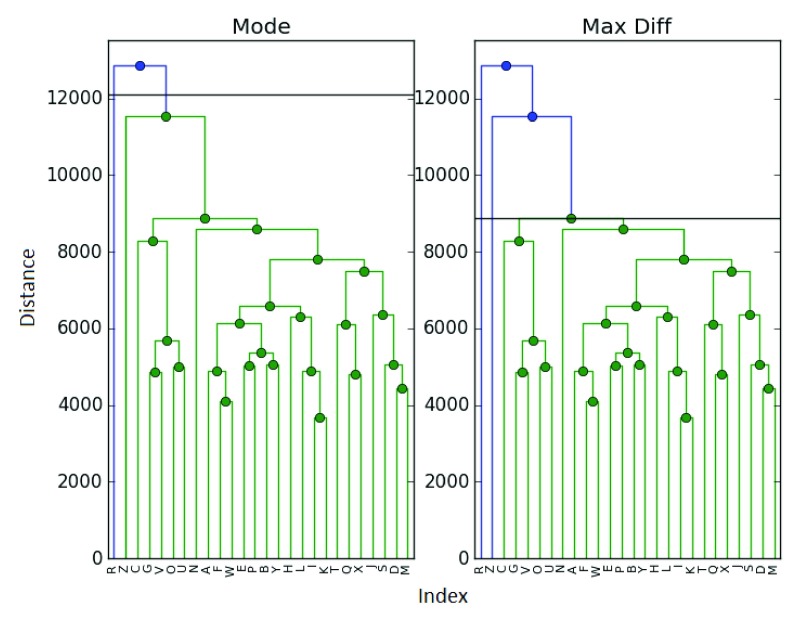
Clustering of the ExpressionSet.

**Figure 5.**
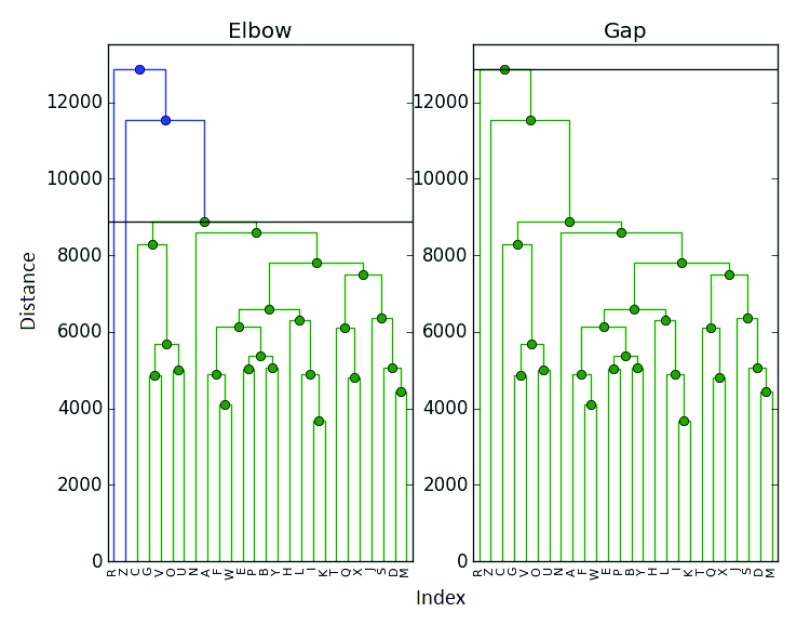
Clustering of the ExpressionSet.

As mentioned in Section 1, our primary motivation for this problem was that of DNA microarray data and gene expression problems. It is also always prudent to test new methods using real data. As such (and to help with reproducibility), we test our methods on the ExpressionSet data from the R package Biobase (Bioconductor) v3.2, ^5^. This is a sample of 26 different elements, with reconstructions presented in Figure 4 and Figure 5.

In this case, the maximum difference and elbow methods were in agreement and selected *k̂ _E_* = *k̂ _D_* = 3 clusters (with samples *R* and *Z* being singleton clusters). The mode, however, chose to add *Z* into the main cluster, producing *k̂ _M_* = 2 final clusters. O the other hand, the gp statistic selects only *k̂ _G_* = 1 cluster.

The author finds both the mode and gap results to be somewhat dubious - but they highlight an important issue. How can we know that a clustering is correct? Even if we examine the dendrogram as we did here, it is likely that in many examples the cutoff point could be debated. In this dataset, we find it more challenging to determine a correct clustering between 2 and 3 clusters - though 3 seems more natural to the author. This calls back to the previously mentioned issue with manual cutoff selection.

## 5 Data mixing

In an effort to improve our new methods, we look at data sampling. Inspired by cross-validation methods, we will randomly sample $M=\frac{N}{2}$ points *L* = 100 times. For each of the *L* samples *j*, we then run our method and get a ${\hat{k}}_{X}^{\left(j\right)}.$ We then set our estimated number of clusters to be

$${\hat{k}}_{X}^{*}=\text{mode}{\;\{{\hat{k}}_{X}^{\left(j\right)}\}}_{j=1}^{L}.\;(10)$$

While this requires running our method *L* times, for *L* = 100, it is still roughly 500 times faster that the gap statistic. Hopefully, this will improve our methods by averaging out any outlying errors or points in our data.

### 5.1 Results

We present results on the same *k* = 2, 3, 4 cluster construction detailed above, each with the same distribution ( Table 7– Table 9). Due to computational times, we did not perform data mixing on the gap statistic. Though given that these methods are much faster even with mixing, we believe that comparing them remains a fruitful exercise. We provide the gap statistic results from Table 1– Table 3 here for convenience.

**Table 7.** Average cluster numbers over *n* = 200 runs.

*k*	${\hat{k}}_{E}^{*}$	k^¯G	${\hat{k}}_{D}^{*}$	${\hat{k}}_{M}^{*}$
2	**2.000**	**2.000**	**2.000**	2.015
3	**3.000**	3.050	**3.000**	3.080
4	**4.000**	4.060	**4.000**	4.065

**Table 8.** Success rate over *n* = 200 runs.

*k*	$S_{E}^{*}$	*S _G_*	$S_{D}^{*}$	$S_{M}^{*}$
2	**1.000**	**1.000**	**1.000**	0.985
3	**1.000**	0.960	**1.000**	0.920
4	**1.000**	0.940	**1.000**	0.935

**Table 9.** Average error size (when wrong) over *n* = 200 runs.

*k*	$E_{E}^{*}$	*E _G_*	$E_{D}^{*}$	$E_{M}^{*}$
2	**0.000**	**0.000**	**0.000**	1.000
3	**0.000**	1.250	**0.000**	1.000
4	**0.000**	1.000	**0.000**	1.000

As shown above, both the elbow method and the maximum difference seem to perfectly capture the simulated data. Perhaps even more surprising, the mode method now has results that are comparable to the gap statistic and not far behind the other methods.

### 5.2 ExpressionSet with mixing

**Figure 6.**
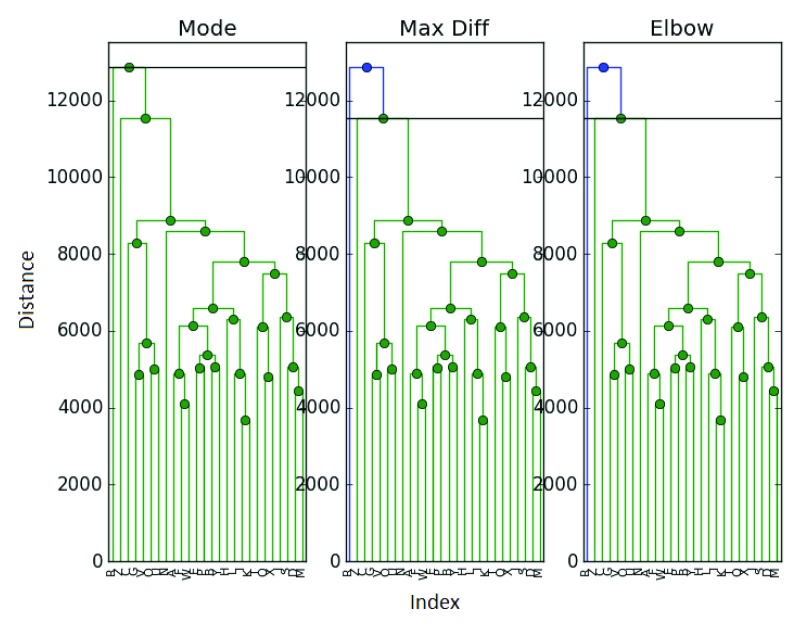
Clustering of the ExpressionSet with mixing.

We now return to the ExpressionSet to see if we can come to a consensus on its clustering. We run the same $M=\frac{N}{2}$ and *L* = 100 mixing as for the simulated data to obtain Figure 6.

With mixing, there are slight differences in clustering. The elbow and maximum difference methods now agree on 2 clusters instead of 3. The mode method agrees with the gap statistic and sets the number of clusters to 1. Again, we find it difficult to argue in favor of 1 cluster, but maintain that 2 or 3 clusters seem viable - with a preference for 3. It is possible we somehow over-mixed the data when working with such a small sample.

### 5.3 LOOCV

**Figure 7.**
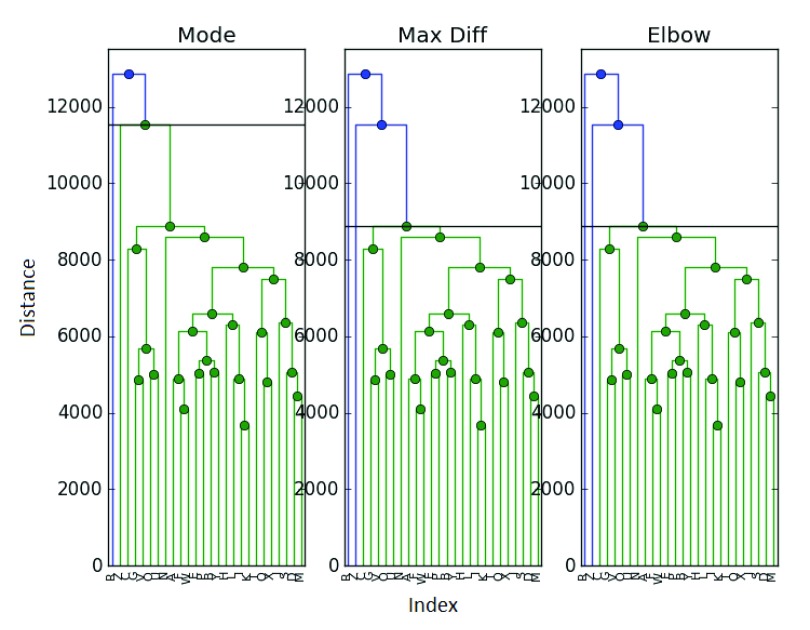
Clustering of the ExpressionSet with “LOOCV”.

Our data mixing procedure resembles leave *N* − *M* out cross validation. In that spirit, let’s examine a method resembling leave one out cross validation (note that computation times will now increase with data size). In our case, this means taking *M* = 1 and removing each data point once (in a sense, *L* = *N*). For each “sampled" set (of *N* − 1 points), we compute the number of clusters and again take the mode of the *L* sample cluster numbers as our estimate. With this we obtain Table 10– Table 12 and Figure 7.

**Table 10.** Average cluster numbers over *n* = 200 runs.

*k*	${\hat{k}}_{E}^{*}$	k^¯G	${\hat{k}}_{D}^{*}$	${\hat{k}}_{M}^{*}$
2	**2.000**	**2.000**	**2.000**	2.660
3	3.040	3.050	**3.000**	4.630
4	3.900	4.060	**4.000**	6.180

**Table 11.** Success rate over *n* = 200 runs.

*k*	$S_{E}^{*}$	$S_{G}$	$S_{D}^{*}$	$S_{M}^{*}$
2	**1.000**	**1.000**	**1.000**	0.465
3	0.960	0.960	**1.000**	0.135
4	0.940	0.940	**1.000**	0.055

**Table 12.** Average error size (when wrong) over *n* = 200 runs.

*k*	$E_{E}^{*}$	$E_{G}$	$E_{D}^{*}$	$E_{M}^{*}$
2	**0.000**	**0.000**	**0.000**	1.234
3	1.000	1.250	**0.000**	1.884
4	2.000	1.000	**0.000**	2.307

While the maximum difference method seems robust in the face of different sampling, it seems that this exercise has revealed some instability in the mode method, which has reverted back to a lackluster performance. To a much lesser extent, the elbow method has some trouble as well. It seems more likely that the choice of sampling parameters could be the cause of the clustering in the Biobase data in Figure 6. More generally, we should look into determining optimal mixing parameters *M* and *L* and/or their impact on these methods.

This mixing method does appear to perform better for the ExpressionSet than the previous choice of mixing parameters, which seems to confirm our hypothesis that there is perhaps an oversampling effect, or something along those lines which must be explored more fully.

## 6 Conclusion

We have developed two new empirical methods for clustering data in a hierarchical framework. While our methods are substantially faster than the existing gap statistic, they do not handle the single-cluster case. In other cases, our maximum difference method is at least comparable to the gap statistic and outperforms the elbow method.

In addition, the use of the data mixing procedure presented here can greatly improve performance (especially for the mode method), leading to the maximum difference method outperforming the other 3. Lastly, these methods can be implemented in a few lines of code and should allow researchers to quickly utilize them at a low computational cost.

In the future we will study the possibility of finding optimal mixing numbers *M* and *L* and the impact of the choice of these parameters of our results. Hopefully, they are related to the instability detected in the mode method when using *M* = 1 in our mixing procedure.

## Data availability

The data referenced by this article are under copyright with the following copyright statement: Copyright: © 2016 Zambelli AE

We provide base code to generate the simulated data used in this paper. The code is written in Python 2.7.



                    from scipy.cluster.hierarchy import linkage
import numpy as np

p = 2 #dimension of the data
n = 100 #number of samples in each cluster
k = 4 #number of clusters

a = np.random.multivariate_normal(\\
    [-3 for _ in range(0,p)],\\
    np.identity(p), size=[n,])
b = np.random.multivariate_normal(\\
    [3 for _ in range(0,p)],\\
    np.identity(p), size=[n,])

#c can be changed to use 2*np.identity(p)
c = np.random.multivariate_normal(\\
    [-3,3], np.identity(p), size=[n,])

#d can be changed to use 0.5*np.identity(p)
d = np.random.multivariate_normal(\\
    [3,-3], np.identity(p), size=[n,])

#X contains the necessary data
X = np.concatenate((a, b, c, d),)

#for reference as to data format usage
Z = linkage(X,'average')
                


In order to access the ExpressionSet data, we use the following R code.



                    library(Biobase)
getClass("ExpressionSet")

#dat contains the necessary data
dat = t(exprs(sample.ExpressionSet))
                

