# Supplementary material for: A data-driven approach to estimating the number of clusters in hierarchical clustering
Source: F1000Res. 2016 Dec 1;5:ISCB Comm J-2809. [Version 1] doi: 10.12688/f1000research.10103.1 (PMC5373427; doi:10.12688/f1000research.10103.1)
Supplement: Peer review history

# Peer review: Referee response for version 1

**DOI:** 10.5256/f1000research.10884.r20844

**Published:** 2017-03-28

**Authors:** Dylan M. Owen

**Affiliations:** 1 Department of Physics, Randall Division of Cell & Molecular Biophysics, Faculty of Life Sciences & Medicine, King's College London, London, UK

Overall I thought it was a very good article with well presented results.

 Where it was slightly lacking was the Introduction - I would have liked more background on the different applications of this type of clustering in the broader scientific field. Are there, for example, applications in physics and cosmology? Ecology and the environment? Cell biology, neurobiology, immunology?? Psychology and sociology?

 A greater comparison to not hierarchical methods would also be appropriate here (or, alternatively in the conclusion). When should such methods be employed in comparison to simple clustering algorithms like Ripley's K-function or DBSCAN for example?

I have read this submission. I believe that I have an appropriate level of expertise to confirm that it is of an acceptable scientific standard.

# Peer review: Referee response for version 1

**DOI:** 10.5256/f1000research.10884.r20087

**Published:** 2017-02-13

**Authors:** Alok Sharma

**Affiliations:** 1 RIKEN Center for Integrative Medical Sciences, Laboratory for Medical Science Mathematics, Yokohama, Japan

This work addresses the concern of clustering analysis. There are mainly two issues in performing clustering: how to locate the clusters in the best possible way, and, how to determine the number of clusters. In most cases the determination of the number of clusters is rather intuitive. Recently some researchers also highlighted this point for hierarchical clustering and other clustering methods. There is a growing need to establish an automated system of estimating the number of clusters accurately for various applications. I hope this work will give due consideration in this field of research.

 Two strategies were considered to find the number of clusters: jump size and maximum difference. It would be better if more theoretical aspect can be given to select parameter *α*, as different values of this parameter can lead to different solution. The same can be argued to maximum difference method which is given as maximum difference plus a constant, again changing this constant can vary the final results. It would be helpful to survey more methods published in this field of research.

I have read this submission. I believe that I have an appropriate level of expertise to confirm that it is of an acceptable scientific standard.

# Peer review: Referee response for version 1

**DOI:** 10.5256/f1000research.10884.r19976

**Published:** 2017-02-07

**Authors:** Xin Zou

**Affiliations:** 1 Shanghai Center for Systems Biomedicine, Shaighai JiaoTong University, Shanghai, China

The authors proposed two simple methods for estimating the number of clusters. However, the results failed to support the motivation.

  

1. The authors criticized  that the current methods are subject to subjective bias. The new methods also rely on human judgement.
2. Computational cost is not a problem nowadays and mathematically the new methods do not show obvious advantages in accelerate the calculation. 

 

 This is not a valuable work for the community and not appropriate to be indexed.

I have read this submission. I believe that I have an appropriate level of expertise to confirm that it is of an acceptable scientific standard, however I have significant reservations, as outlined above.
